# Neutralizing Anti-SARS-CoV-2 Antibody Titer and Reported Adverse Effects, in a Sample of Italian Nursing Home Personnel after Two Doses of the BNT162b2 Vaccine Administered Four Weeks Apart

**DOI:** 10.3390/vaccines9060652

**Published:** 2021-06-15

**Authors:** Alberto Modenese, Stefania Paduano, Annalisa Bargellini, Rossana Bellucci, Simona Marchetti, Fulvio Bruno, Pietro Grazioli, Roberto Vivoli, Fabriziomaria Gobba

**Affiliations:** 1 Department of Biomedical, Metabolic and Neural Sciences, University of Modena & Reggio Emilia, 41125 Modena, Italy; stefania.paduano@unimore.it (S.P.); annalisa.bargellini@unimore.it (A.B.); fabriziomaria.gobba@unimore.it (F.G.); 2 Laboratorio Analisi TEST SrL, 41121 Modena, Italy; bellucci.rossana@laboratoriotest.it (R.B.); marchetti.simona@laboratoriotest.it (S.M.); vivoli.roberto@laboratoriotest.it (R.V.); 3 Fondazione Scarpari Forattini Onlus, 46020 Schivenoglia, MN, Italy; fbruno@scarpari.it (F.B.); graziolipietro@me.com (P.G.)

**Keywords:** SARS-CoV-2, COVID-19, healthcare workers, vaccines, antibody titer, adverse effects, health surveillance, BNT162b2

## Abstract

Background: The immunization of healthcare workers (HCWs) plays a recognized key role in prevention in the COVID-19 pandemic: in Italy, the vaccination campaign began at the end of December 2020. A better knowledge of the on-field immune response in HCWs, of adverse effects and of the main factors involved is fundamental. Methods: We performed a study on workers at a nursing home in Northern Italy, vaccinated in January–February 2021 with two doses of the BNT162b2 vaccine four weeks apart, instead of the three weeks provided for in the original manufacturer protocol. One month after the second dose, the serological titer of IgG-neutralizing anti-RBD antibodies of the subunit S1 of the spike protein of SARS-CoV-2 was determined. The socio-demographic and clinical characteristics of the subjects and adverse effects of vaccination were collected by questionnaire. Results: In all of the workers, high antibody titer, ranging between 20 and 760 times the minimum protective level were observed. Titers were significantly higher in subjects with a previous COVID-19 diagnosis. Adverse effects after the vaccine were more frequent after the second dose, but no severe adverse effects were observed. Conclusions: The two doses of the BNT162b2 vaccine, even if administered four weeks apart, induced high titers of anti-SARS-CoV-2 neutralizing IgG in all the operators included in the study.

## 1. Introduction

The vaccination campaign aimed at preventing the infectious risk related to SARS-CoV-2 in healthcare workers (HCWs) in Italy began on 27 December 2020 [1], and the majority of the workers who joined the campaign received two doses of the vaccine by the end of February. Currently in Italy, as elsewhere in Europe, four vaccines are approved by the national authority (Agenzia Italiana del Farmaco, AIFA, based in Rome, Italy): two mRNA-based vaccines, the BNT162b2 and the mRNA-1273 vaccines, and two viral vector vaccines, i.e., the ChAdOx1-S and the Ad26.COV2.S vaccines [2]. The two mRna-based vaccines have been used in Italy since the beginning of the campaign involving HCWs. Both require two doses administered three weeks apart for the BNT162b2 and four weeks apart for the mRNA-1273, according to the original protocol, resulting in a protective effect after 21 days for BNT162b2 and after 28 days for mRNA-1273 [2].

In order to evaluate the immune response to the vaccines, the quantification of the serum of the neutralizing type G immunoglobulins produced after the second dose of the vaccines should be determined [3]. These antibodies are specific to the receptor binding domain (RBD) of the subunit S1 of the spike protein of SARS-CoV-2, and therefore they are called anti-RBD antibodies [3]. An intense immunological response to the vaccines may be associated with higher reporting of adverse effects after the administration of the dose(s), e.g., reactions involving asthenia, fever, arthralgia, myalgia and other symptoms [4].

As for other vaccines, various factors can influence the immunological response. As examples, a reduced response can be expected in cases of immunosuppression [5], while increased antibody production can be related e.g., to a previous exposure to the infectious agent that the vaccine intends to prevent [6]. Individual factors, such as smoking, obesity and hypertension, can also possibly influence the response [7].

The objective of this work is to evaluate the response to anti-SARS-CoV-2 vaccination in a sample of HCWs in a nursing home in Northern Italy using two doses of the BNT162b2 anti-SARS-CoV-2 vaccine administered four weeks apart (instead of the three weeks indicated in the original protocol) and the main factors, including previous SARS-CoV-2 infection, occupational and socio-demographic factors, related to the antibody titer. In the same sample, we also studied the type, frequency and duration of the adverse effects after the first and the second dose of the vaccine and the possible associated factors.

## 2. Materials and Methods

### 2.1. Study Context and Population

We performed an observational study in a group of workers employed in a nursing home in Northern Italy, hospitalizing about 80 inpatients, mainly elderly, with different degrees of autonomy in daily activities. The employees received two doses of the anti-SARS-CoV-2 BNT162b2 vaccine between the 12th of January and the 17 of February 2021. It should be noted that all the inpatients were also vaccinated in the same period. The only inclusion criteria for this study were:

- being an employee of the nursing home at work during the period January–February 2021
- being vaccinated with the BNT162b2 anti-SARS-CoV-2 vaccine
- having completed the two-dose cycle with an interval of four weeks between the first and the second dose.

The interval of four weeks between the first and the second dose of the BNT162b2 vaccine, instead of the three weeks recommended by the manufacturer, was not intentional; it was a consequence of the vaccines’ supply issues, involving all of Italy, especially during the first months of the vaccination campaign, that causing delays in the delivery of the vaccine’s doses.

No exclusion criteria were defined based on the specific job tasks: accordingly, the participation in the study was offered to all the workers employed in the clinic, including HCWs (i.e., nurses, assistant nurses, physicians and others) as well as administrative and cleaning service personnel, kitchen staff and maintenance workers.

For all the personnel who voluntarily agreed to participate, a written informed consent was collected. The study was approved by the institutional review board with the code 776/2020/SPER/UNIMO SIRER ID 540.

### 2.2. Quantification of Neutralizing Antibodies in the Serum

For all the subjects, four weeks after the second dose of the vaccine, a blood sample was collected to determine the titer of IgG-neutralizing anti-RBD antibodies of the subunit S1 of the spike protein of SARS-CoV-2. The EUROIMMUN Anti-SARS-CoV-2 QuantiVac ELISA (IgG) test was applied: this methodology quantifies the concentration of IgG antibodies against the S1 antigen (including RBD) of SARS-CoV-2 in a broad linear range, using a 6-point calibration curve [8]. This method has proved to have excellent correlation with the WHO standards [9]. The antibody concentration is expressed as relative units per milliliter (RU/mL), and, according to the data sheet of the manufacturer, the interpretation of the results is as follows: sample is negative for antibody titer <8 RU/mL, positive with value ≥ 11 RU/mL, while values between 8 and 11 are of doubtful interpretation. The manufacturer also indicates how to convert RU to binding antibody units (BAU), which are the units of measure adopted for our analysis, obtained by multiplying the RU values by the factor 3.2 [8]. Accordingly, the non-protective and protective antibody threshold levels are as follows: lower than 26 BAU/mL and ≥36 BAU/mL respectively, with doubtful interpretation between 26 and 35 BAU/mL.

### 2.3. Collection of Information from the Study Participants

A self-administrated questionnaire was collected for all of the subjects, including questions on:

(a). socio-demographic (age, sex) and anthropometric (height, weight) information, as well as on smoking and alcohol consumption habits
(b). occupational anamnesis including an investigation of job tasks and of the eventual performance of nightshifts at work
(c). a pathological anamnesis, investigating in particular a previous SARS-CoV-2 infection and medical conditions, because immunosuppression is able to influence the immune response to the vaccine
(d). the occurrence and duration of adverse effects after the first and the second anti-SARS-Cov-2 vaccine doses, based on a list of effects reported in the Vaccine Surveillance Report of the AIFA [10] and on a recent study [11], including both local (pain and/or redness in the injection site) and general symptoms (fever, asthenia/fatigue, muscle aches, anaphylaxis and other).

### 2.4. Statistical Analysis

Logarithmic transformation was used to normalize anti-SARS-CoV-2 neutralizing IgG; square root transformation was used to normalize number of adverse effects, and the results are expressed as median and range. The chi-square test, *t* test, and one-way analysis of variance (ANOVA) with the Bonferroni test were applied whenever necessary. The data collected were analyzed using STATA Software (release 15, StataCorp, College Station, TX, USA). Regarding statistical significance, a *p* value < 0.05 was considered significant.

## 3. Results

### 3.1. General Characteristics of the Study Population and Anti-SARS-CoV-2 Antibody Titer in the Vaccinated Subjects

All but two of the 76 employees of the nursing home (97.4%) agreed to participate. Accordingly, the sample was composed of 74 workers, mean aged 48.4 years (Standard Deviation—SD = 13.4); the large majority (81.1%) were females.

In all of the workers, two doses of the BNT162b2 vaccine were administered, the second dose four weeks after the first. The median titer of the anti-SARS-CoV-2 neutralizing IgG against the subunit S1 of the spike protein in the serum was 4892 BAU/mL, with a range 764–27,600 BAU/mL. Results show that four weeks after the second dose of the vaccine, in all subjects the titer was largely above the limit considered to be protective (>36 BAU/mL).

In Table 1, we present the values obtained in the whole sample, and the differences among the median values of the titers in the group stratified according to the following characteristics: sex, age classes, BMI, smoking habit (yes/no), job category (workers with high infectious risk, including HCWs, such as nurses, nurse assistants, physicians versus workers with medium or low infectious risk, including other HCWs, e.g., physiotherapists, cleaning and kitchen personnel, technical and administrative workers), nightshifts at work (yes/no) and anti-influenza vaccination at the end of 2020. Moreover, we also evaluated differences in the median antibody titer according to the reporting of a previous COVID-19 diagnosis (Table 1).

Considering the distribution according to job activity, about half of our sample (45%) was composed of HCWs engaged in patient care (nurses, assistant nurses and a physician) that can be considered at high COVID-19 risk (high infectious risk). Another 18% of the operators were HCWs engaged in activities with reduced contacts with the patients (especially during the hardest periods of the pandemic): physiotherapists, occupational therapists and others; these operators were classified “at medium risk of infection”. Non-HCWs included cleaning personnel (12%) and kitchen personnel (8% of the sample), also classified at medium infectious risk, while other technical and administrative personnel (17.6%) were evaluated “at low infectious risk”. No significant differences were observed between HCWs and non-HCWs (data not presented) but, when comparing job categories according to the risk level, high-risk operators proved to have significantly higher neutralizing anti-SARS-CoV-2 antibodies titer (*p* = 0.027) compared to the medium/low risk workers (Table 1).

Considering now the differences according to the occurrence of a previous SARS-CoV-2 infection, 31 subjects (41.9% of the whole sample) reported a COVID-19 diagnosis before the vaccine: in this group, the median IgG level result was significantly higher compared to the workers without a previous infection: 6856 (range = 1310–20,300) versus 3746 (range = 764–27,600) BAU/mL, *p* = 0.006 (Table 1). Considering the reported date of infection, of the 31 COVID-19 cases in our sample, 18 workers were diagnosed during the first wave of the pandemic in Italy, between March and May 2020, while 9 were diagnosed during the second wave, between November 2020 and January 2021. Moreover, 4 workers have been infected with SARS-CoV-2 two times, respectively during the first and the second wave of the pandemic. We did not find any statistically significant difference in the median anti-SARS-CoV-2 antibody titer among these groups of subjects (data not shown). Only one of the workers (i.e., one of the four who reported having been diagnosed with SARS-CoV-2 two times) got the infection after the vaccination, and in particular, seven days after the first dose. The infection was asymptomatic, and the worker could go back to work after ten days after the diagnosis, with a negative swab, and was able to complete the second dose of the vaccination four weeks after the first dose as the rest of the group did.

### 3.2. Analysis of the Adverse Effects Reported after the Anti-SARS-CoV-2 Vaccine

**Figure 1:**
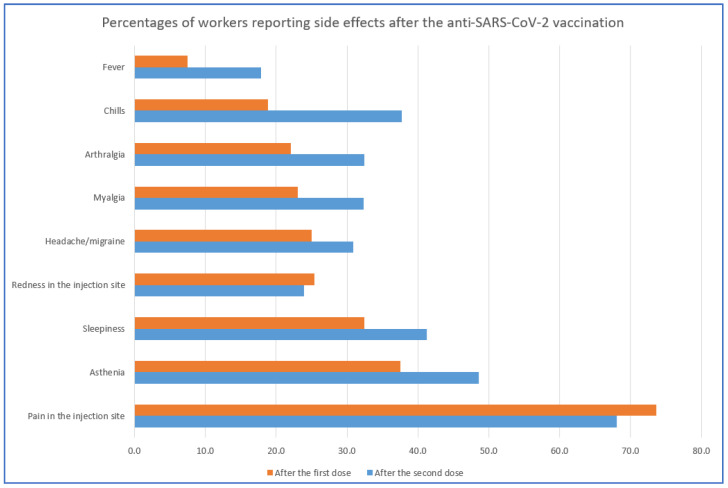
Percentage of workers reporting adverse effects after the first (red bar) and the second (blue bar) dose of the BNT162b2 anti-SARS-CoV-2 vaccine. NB: data on other adverse effects (including anaphylaxis, syncope, dyspnea, diarrhea, erythema, abdominal pain, itch, vertigo and taste alterations) with a frequency <8% are not shown here.

According to the answers to the questionnaire, the most frequent symptom reported after the vaccination was a local one: pain sensation at the injection site, reported by 73.6% and the 68.1% of the sample, respectively, after the first and after the second dose of the vaccine (Figure 1). Another common local symptom was redness in the injection site, present in about 1/4th of the respondents and slightly more frequent after the first dose. The most frequent general symptoms were asthenia and sleepiness: a proportion ranging from 1/3rd up to a half of the respondents reported at least one of these two symptoms, with a higher proportion after the second dose. All the other adverse effects were also reported more frequently after the second dose, with maximum values involving 37.7% of the sample for chills, 32% for both myalgia and arthralgia, 31% for headache/migraine and 18% for fever (Figure 1). Less frequently observed symptoms were diarrhea, erythema, abdominal pain, itch and vertigo, with percentages of subjects ranging between 6 and 8% of the overall sample after the second dose and lower frequencies after the first dose. Finally, three subjects, two after the second dose and one after the first, reported taste alterations, a symptom quite typical of SARS-CoV-2 infection.

Anaphylactic reactions, syncope and dyspnea are among the most severe adverse effects described after the vaccine [12]. In our sample, anaphylaxis and syncope were reported by one only worker, without previous SARS-CoV-2 infection. An episode of dyspnea was referred by two workers with a previous diagnosis of COVID-19: one reported the effect after the first dose only, while the other one reported it after the second dose.

We also evaluated the occurrence of the symptoms after vaccination in the workers with a previous COVID-19 diagnosis versus respondents without a previous SARS-CoV-2 infection (Table 2). After the first vaccine dose, local effects (redness and especially pain in the injection site) were more frequent, even if not significantly, in the COVID-19 group, reaching percentages up to 80% (Table 2). Also other symptoms (with the exception of fever) were more frequent in the COVID-19 group, with the highest occurrence observed for asthenia, involving 51.7% of the workers, and the lowest for chills (20%); although only for asthenia and arthralgia, reported respectively by 51.7% and 35.7% of the COVID-19 sub-group compared to 27.9% and 12.5% of the non-COVID-19 subjects, is the difference statistically significant (*p* < 0.05).

After the second dose, the differences were flattened, and only arthralgia, headache/migraine, myalgia and chills resulted more frequently in the COVID-19 group, while asthenia and sleepiness were slightly more frequent among subjects without a previous diagnosis of SARS-CoV-2 infection; none of the differences is significant (Table 2).

Interestingly, only for fever was the proportion of respondents approximately the same in both groups: after the first dose, 7.1% and 7.7% in the COVID-19 group and non-COVID-19 respondents, respectively; then, those groups’ results more than doubled but were still superimposable (17.9% in both) after the second dose (Table 2).

Considering the duration of the symptoms after the first and second dose, in about 50% of the respondents at least one adverse effect persisted for more than 24 h, without a difference between the two doses. In 20 subjects (27%), at least one adverse effect lasted for more than 48 h. Only two workers required, after the first dose, respectively, one and four days-off work, and neither of them reported a previous COVID-19 diagnosis. Six workers after the second dose had symptoms severe enough to require a day or more off work. Among these six workers, two did not have a history of SARS-CoV-2 infection, while four did. Of the six workers, four required two days off (three of them with COVID-19 history, and one without); one worker with no COVID-19 history had only one day off work, and one subject did not report the length of the absence from work.

We further analyzed the adverse effects lasting >24 h, and the possible differences between COVID-19 and non-COVID-19 groups (Table 3). We found that, after both doses of the vaccine, in the workers with a previous COVID-19 diagnosis, the median number of these longer-lasting effects was higher, even if the difference is significant (*p* < 0.05) only for “general adverse effect” after the second dose (Table 3).

Lastly, we tested the hypothesis of a possible relationship between anti-SARS-CoV-2 neutralizing antibody titer after the vaccine and the reporting of adverse effects. The results, shown in Table 4, indicate that only after the first vaccine dose, and only for the general adverse effects, the IgG levels were significantly higher (*p* < 0.05), while in all the other cases the occurrence of symptoms and the neutralizing antibody titer seem apparently unrelated (Table 4).

## 4. Discussion

According to the obtained results, one month after the second of two doses of the BNT162b2 vaccine, in all the nursing home employees enrolled in the study, the median titer of the anti-SARS-CoV-2 neutralizing IgG against the subunit S1 of the spike protein in the serum was 4892 (range 764–27,600 BAU/mL), i.e., more than 136 times the minimum level considered to be protective (>36 BAU/mL). Considering individual values, the lowest measured titer was 764 BAU/mL, i.e., more than 20 times the minimum level, and the highest was 27,600 BAU/mL (more than 760 times). As a whole these results are coherent with some data recently published by other groups [13,14,15].

Interestingly, these results were obtained with two doses of the vaccine administered four weeks apart, instead of the three weeks required in the original protocol of the manufacturer [16], supporting the hypothesis that a longer lag period between the two doses (at least, up to one week) does not interfere with an adequate response.

The titer of anti-SARS-CoV-2 antibodies was proved to be significantly higher in operators with a previous COVID-19 diagnosis (Table 1): the frequency of the disease was quite high among the workers, affecting 42% of them, and it should be noted that, in 2020, the nursing home had been deeply impacted by the pandemic, with about 80 cases registered among the inpatients. In the year 2021 and with the beginning of the vaccination campaign, things have progressively improved, with only 3 cases among inpatients, and only 1 new case among the workers, which interestingly occurred one week after the first dose of the vaccination; the worker fully recovered in 10 days with no symptoms of the infection.

Considering other factors possibly affecting the levels of anti-SARS-CoV-2 antibodies, in our study sex, age, obesity and smoking habit apparently did not significantly affect the titer, which is different than other observations [7].

Regarding job characteristics, HCWs with a higher infectious risk, i.e., nurses, nurses’ assistants and physicians, showed a higher neutralizing antibody level compared to those workers at medium (i.e., other HCWs like physiotherapists, cleaning and kitchen personnel) or at low infectious risk (technical and administrative personnel). Nightshifts seemed to not influence the response (Table 1).

Considering now the adverse effects (Table 2, Figure 1), in general, the symptoms were more frequently reported after the second dose of the vaccine, possibly as a consequence of a stronger immunological response. The most frequent effect was a local one (pain in the injection site), involving about 70% of the workers after both doses. Among the general adverse effects, the most frequent were asthenia and sleepiness, reported by about 1/3rd and about a half of the operators, respectively, after the first and after the second dose of the vaccine, while myalgia, arthralgia, chills and headache/migraine appeared in about 20% and 40% of the workers, respectively, after the two doses. These results are in agreement with some preliminary data of other authors [17,18].

## 5. Conclusions

The two doses of the BNT162b2 vaccine, even if administered four weeks apart, induced high titers of anti-SARS-CoV-2 neutralizing IgG against the subunit S1 of the spike protein in all the respondents included in the study, ranging between 20 and 760 times the minimum level considered to be protective. No severe adverse effects were observed in the examined workers, and in only 27% of the subjects at least one adverse effect lasted for more than 48 h; the prevalence of all the general symptoms was higher after the second dose, especially sleepiness and asthenia, involving more than 1/3rd of subjects. Lastly, in general, the prevalence of symptoms, and of symptoms lasting more than 24 h, tended to be higher in workers who reported a previous COVID-19 diagnosis, even if the difference was significant only for general adverse effects.

## Figures and Tables

**Table 1:** Median values and range of neutralizing antibodies against SARS-CoV-2, measured 4 weeks after two doses of the BNT162b2 vaccine administered 4 weeks apart, in 74 workers of an Italian nursing home grouped according to the main individual and occupational characteristics.

Characteristics of the Studied Subjects		% (N) *	Anti-SARS-CoV-2 IgG (BAU/mL) MD (Range)	*p* Value
Sex	Males	18.9 (14)	3335 (764–17,300)	0.198
	Females	81.1 (60)	5285 (830–27,600)	
Age class	≤30	12.2 (9)	7050 (3272–13,749)	0.165
	31–40	16.2 (12)	3086.5 (1824–11,390)	
	41–50	18.9 (14)	4820 (1243–20,300)	
	51–60	31.1 (23)	4821 (830–15,474)	
	>60	21.6 (16)	6542.5 (764–27,600)	
BMI	<25	53.4 (39)	4821 (830–20,300)	0.819
	25–29.9	37.0 (27)	6783 (764–17,300)	
	≥30	9.6 (7)	4557 (1794–14,834)	
Smoking habit	Non–smokers	77.0 (57)	4557 (764–27,600)	0.554
	Smokers	23.0 (17)	6783 (1824–13,551)	
Job category	High infectious risk HCWs **	44.6 (33)	6783 (1504–27,600)	0.027
	Medium/low infectious risk ***	55.4 (41)	4150 (764–20,300)	
Nightshifts at work	No	77.8 (56)	4202 (764–20,300)	0.124
	Yes	22.2 (16)	6953 (2508–27,600)	
Anti-influenza vaccine	No	60.8 (45)	5062 (830–27,600)	0.465
	Yes	39.2 (29)	4497 (764–17,300)	
Previous COVID-19 diagnosis	No	58.1 (43)	3746 (764–27,600)	0.006
	Yes	41.9 (31)	6856 (1310–20,300)	

MD = median, BAU/mL (binding antibody units per milliliter), HCWs (healthcare workers), BMI (body mass index). * The percentages were calculated excluding missing values. ** nurses, nurses’ assistants, physicians; *** other HCWs, such as physiotherapists and occupational therapists, cleaning and kitchen personnel, technical and administrative personnel.

**Table 2:** Frequency of main adverse effects reported by the workers after the first and the second dose of the BNT162b2 anti-SARS-CoV-2 vaccine.

Adverse Effect Reported	Vaccination Dose	Workers with the Adverse Effects% (N) *	Workers with the Adverse Effects in the COVID-19 Group% (N) *	Workers with the Adverse Effects in the Non-COVID-19 Group % (N) *	*p*-Value
Redness in the injection site	1st	25.4 (17)	33.3 (9)	20.0 (8)	0.219
	2nd	23.9 (16)	25.9 (7)	22.5 (9)	0.747
Pain in the injection site	1st	73.6 (53)	80.0 (24)	69. 0 (29)	0.299
	2nd	68.1 (49)	73.3 (22)	64.3 (27)	0.417
Fever	1st	7.5 (5)	7.1 (2)	7.7 (3)	0.933
	2nd	17.9 (12)	17.9 (5)	17.9 (7)	0.992
Asthenia	1st	37.5 (27)	51.7 (15)	27.9 (12)	0.041
	2nd	48.6 (35)	48.3 (14)	48.8 (21)	0.963
Myalgia	1st	23.1 (15)	29.6 (8)	18.4 (7)	0.291
	2nd	32.3 (21)	37.0 (10)	28.9 (11)	0.492
Arthralgia *	1st	22.1 (15)	35.7 (10)	12.5 (5)	0.023
	2nd	32.4 (22)	42.9 (10)	25.0 (10)	0.121
Headache/migraine	1st	25.0 (17)	35.7 (10)	17.5 (7)	0.088
	2nd	30.9 (21)	39.3 (11)	25.0 (10)	0.210
Chills	1st	18.8 (13)	20.0 (6)	17.9 (7)	0.829
	2nd	37.7 (26)	40.0 (12)	35.9 (14)	0.727
Sleepiness	1st	32.4 (22)	41.4 (12)	25.6 (10)	0.170
	2nd	41.2 (28)	34.5 (10)	46.2 (18)	0.333

* The percentages were calculated excluding missing values. NB: data on other adverse effects (including anaphylaxis, syncope, dyspnea, diarrhea, erythema, abdominal pain, itch, vertigo and taste alterations) with a frequency <8% are not shown here.

**Table 3:** Adverse effects (all versus general effects, i.e., excluding local effects) lasting more than 24 h after the first and second dose of BNT162b2 anti-SARS-CoV-2 vaccine in the workers with and without a previous COVID-19 diagnosis.

Previous COVID-19	After 1st Dose				After 2nd Dose			
	All the Adverse Effects		General Adverse Effects *		All the Adverse Effects		General Adverse Effects *	
	Workers % (N)	Number of Effects **	Workers % (N)	Number of Effects **	Workers % (N)	Number of Effects **	Workers % (N)	Number of Effects **
Yes	44.1 (26)	1 (0–11)	47.5 (19)	0 (0–10)	43.3 (26)	1 (0–10)	38.0 (19)	2 (0–9) ***
No	55.1 (33)	0 (0–5)	52.5 (21)	0 (0–5)	56.7 (34)	0 (0–9)	62.0 (31)	0 (0–8) ***

* i.e., without local effects (including pain and redness in the injection site); ** Median value (range); *** *p* = 0.045.

**Table 4:** Median levels of anti-SARS-CoV-2 neutralizing antibodies (IgG) in operators without and with adverse effects (all versus general effects, excluding local effects), after the first and the second dose of the BNT162b2 anti-SARS-CoV-2 vaccine.

% (n) of Workers with/without the Adverse Effects & Median (Range) of Neutralizing Anti-SARS-CoV-2 IgG (BAU/mL)							
After 1st Dose				After 2nd Dose			
All the Adverse Effects% (N)		General Adverse Effects (Not Considering Local)% (N)		All the Adverse Effects% (N)		General Adverse Effects (Not Considering Local)% (N)	
Yes	No	Yes	No	Yes	No	Yes	No
79.7 (59) 4821 (1243–27,600)	20.3 (15) 5087 (764–13,749)	54.0 (40) 5223 (1504–27,600)	46.0 (34) 4256.5 (764–13,749)	81.1 (60) 4699.5 (830–27,600)	18.9 (14) 5651.5 (764–11,638)	67.6 (50) 4699.5 (830–27,600)	32.4 (14) 5361 (764–17,300)
n.s.		*p* = 0.017		n.s.		n.s.	

## Data Availability

The data presented in this study are available on request from the corresponding author. The data are not publicly available due to restrictions according to privacy and ethical reasons.
